# Circulating direct infusion MS and NMR metabolomic profiles of post-gonadectomy kittens with or without additional dietary choline supplementation

**DOI:** 10.1017/S0007114522003385

**Published:** 2023-08-14

**Authors:** Hannah Godfrey, Alexandra Rankovic, Caitlin E. Grant, Sarah K. Abood, Anna Kate Shoveller, Marica Bakovic, Adronie Verbrugghe

**Affiliations:** 1 Department of Clinical Studies, Ontario Veterinary College, University of Guelph, Guelph, ON N1G 2W1, Canada; 2 Department of Biomedical Sciences, Ontario Veterinary College, University of Guelph, Guelph, ON N1G 2W1, Canada; 3 Department of Animal Biosciences, Ontario Agricultural College, University of Guelph, Guelph, ON N1G 2W1, Canada; 4 Department of Human Health and Nutritional Sciences, College of Biological Sciences, University of Guelph, Guelph, ON N1G 2W1, Canada

**Keywords:** Spay/neuter, Direct infusion MS, Quantitative NMR spectroscopy, One-carbon metabolism, Feline obesity, Nutraceuticals and functional foods, Feline nutrition

## Abstract

Choline is beneficial for energy metabolism and growth in various species. Choline may work similarly in kittens at risk of obesity. Direct infusion MS (Di-MS) and NMR spectroscopy were used to investigate the metabolomic signatures of kittens supplemented with or without additional dietary choline for 12 weeks. Fifteen intact male kittens consumed a base diet (3310 mg choline/kg DM) to their daily metabolisable energy requirement (DER) over an 11-week acclimation. Kittens were gonadectomised and assigned, based on body weight, to the base diet (CONTROL, *n* 7) or the base diet with 300 mg/kgBW^0·75^ additional choline as choline chloride (CHOLINE, *n* 8) and offered three times their individual energy requirement divided into three meals. At weeks −1 and 12, fasted blood was sampled and serum analysed for 130 metabolites via Di-MS and fifty-one metabolites via NMR spectroscopy. Changes in fasted metabolites were assessed using a repeated-measures GLIMMIX procedure with time and group as fixed effects, and time as a repeated measure. Metabolites of one-carbon metabolism and lipids increased, and medium-chain acyl carnitines decreased from week −1 to 12 for CHOLINE (*P* < 0·05), but not CONTROL (*P* > 0·05). Increases in amino acid, biogenic amine and organic compound concentrations were observed in both groups (*P* < 0·05). The results suggest impacts of dietary choline at greater intakes than currently recommended on one-carbon metabolism and fatty acid oxidation, and these may promote healthy growth in post-gonadectomy kittens.

Obesity in domestic cats is a major health risk leading to further complications, which impact both the quality and quantity of life^(1,2)^. Subsequently, treatment and prevention of obesity has been a focus for both the veterinary community and pet food industry. Gonadectomy in cats is considered a risk factor for obesity^(3–5)^. Gonadectomy is recommended for cats due to its benefits for population control, in curbing behavioural patterns^(6)^, specifically in males, and reducing risk of reproductive disorders such as leukaemia^(7)^. However, the increased food intake, higher body weights (BW) and higher percent in body fat that occur post-gonadectomy may lead to an obese state^(4,5,8,9)^.

Due to the complexity of metabolic pathways, research that employs a dietary intervention to minimise or prevent obesity has recently been using metabolomic approaches to characterise the physiological changes that occur among dietary treatments and over time^(10,11)^ and develop targeted hypotheses for more precise subsequent experimental designs.

A nutrient of interest is choline: an essential nutrient and water-soluble quaternary ammonium compound. Choline is involved in three major areas. First, as acetylcholine, choline is involved in pathways for memory and cognition, signalling muscular movement, and endocrine system regulation^(12)^. Second, choline can be converted to betaine where it acts as a methyl donor for methionine re-methylation from homocysteine. Methionine plays an important role in DNA methylation where it is involved in gene regulation that influences metabolism and growth^(12,13)^. Finally, as phosphatidylcholine (PC), choline is involved in cell signalling and membrane transport, and integrity of the cell membrane^(12–14)^. VLDL require PC in order to be secreted from hepatocytes and into bile. Choline deficiency results in liver dysfunction and fatty liver, which is life-threatening, due to an accumulation of TAG from a reduced secretion of VLDL^(15–17)^. In the muscle, PC can alter intracellular lipid metabolism. In choline deficiency, there is muscle lipid accumulation of diacylglycerides and a reduction of TAG^(12–14,16,18)^. For kittens, choline recommendations are established from studies in which the requirement was measured by reversing or preventing fatty liver^(19,20)^. However, there may be additional benefits of choline, and the requirement for choline may be greater when evaluating alternative outcomes. For example, in humans and other species, choline deficiency also results in atherosclerosis and neurological disorders. These disorders have not been reported or investigated in cats in response to a choline deficiency; however, similar to other species, a choline deficiency is documented to impair growth^(19,21)^.

Due to its important role in lipid metabolism, specifically in the liver, researchers have investigated the role of choline supplementation on growth, body composition and weight loss^(19,20)^. However, a primary focus has been on improving carcass composition in livestock species^(21–24)^. In companion animals, multiple supplements have been assessed for obesity prevention or treatment, including l-carnitine^(25)^. There is a significant lack of research examining the role of choline and lipid metabolism in companion animals, specifically in cats. From the current research in livestock, additional choline supplementation results in increased lean muscle mass and reduced body fat mass^(26–29)^, making dietary choline a promising dietary intervention that may aid in preventing obesity in gonadectomised kittens.

Metabolomic signatures have been useful in understanding how choline can impact both one-carbon and lipid metabolism. A study investigating high-fat diets (45 % ME from fat) over a 12-week period in rats applied metabolomics to assess hepatic metabolism^(30)^. Lower methionine and greater choline were noted in the liver of rats fed high-fat diets, suggesting that choline-driven one-carbon metabolism in the liver was reduced in insulin resistance and models of hepatic steatosis^(30)^. Likewise, metabolomics is being employed to understand acylcarnitine metabolism and how that plays a role in the development of obesity^(25,26,31,32)^. Metabolomic approaches could prove useful when applied to dietary intervention studies and improve the overall understanding of how supplementation impacts metabolic pathways. For example, supplementation of choline and betaine altered the metabolomic profile of plasma metabolites in mice. Specifically, one-carbon metabolism appeared to be upregulated in supplemented mice, such that betaine and methionine levels were greater^(33)^. Therefore, we hypothesise that greater choline intake alters metabolomic profiles, specifically one-carbon and lipid metabolism, in kittens after gonadectomy.

Using metabolomic data from direct infusion MS (Di-MS) and NMR technologies, this study aimed to measure changes in biochemical pathways that may be impacted by additional dietary choline in post-gonadectomy kittens. It was predicted that choline supplementation would increase one-carbon metabolism via betaine, thus increasing serum concentrations of metabolites of one-carbon metabolism, such as betaine, dimethylglycine, sarcosine, and methionine, and potentially reduce reliance on serine and folate from the re-methylation of methionine via the folate cycle. Furthermore, it was predicted that there would be an observed increase in serum concentrations of PC and subsequently increases in lysophosphatidylcholines (LPC) and sphingomyelin (SM) concentrations after choline supplementation. These results will help to understand the metabolic signature of choline supplementation in post-gonadectomy kittens.

## Materials and methods

### Animals

As previously described^(34)^, sixteen intact male kittens aged 12–13 weeks old (Marshall’s Bio Resources) participated in the study. BW was a mean (±sem) of 3·85 ± 0·067 kg before gonadectomy, when kitten where 24–25 weeks old. All kittens had a body condition score between 4 and 6 on a nine-point scale^(35)^. A physical examination, serum biochemistry profile and complete blood count were conducted, and all animals were deemed healthy prior to the study. Experimental procedures were approved by the University of Guelph Animal Care Committee (AUP #4118) and were in accordance with national and institutional guidelines for the care and use of animals in research as described below.

### Housing

Cats were housed in an indoor free-living environment (23 ft × 19 ft), enriched using multiple scratching posts, cat trees, hiding boxes, perches, beds and toys, at the University of Guelph, Ontario Agricultural College, Animal Biosciences Cattery (Guelph, ON). Voluntary brushing, petting and play for a maximum of 2 h per d for 5 d by familiar individuals was provided. *Ad libitum* water was available in bowls around the room and as a dripping tap. Temperature ranged from 19·6°C to 25·3°C, and mean humidity was 48·4 %. A 12-h light and dark cycle was maintained with lights on at 07·00 h and off at 19.00 h. Daily cleaning and sanitisation were followed, and litter boxes were scooped twice daily. Designated animal care attendants provided daily animal checks to ensure all animals were healthy and not exhibiting signs of stress for the duration of the study.

### Diet and choline supplementation

A commercially available extruded diet formulated for growth according to the Association of American Feed Control Officials (AAFCO)^(36)^ was used as the base diet for the study (Nutram Sound Balanced Wellness® Kitten Food Chicken Meal and Salmon Meal Recipe, Elmira Pet Products, Elmira, ON, Canada). Proximate, vitamin and amino acid analyses were described earlier^(34)^ and presented in Supplementary Table S1. Kittens were provided their daily metabolisable energy requirement for growth during an 11-week acclimation period using the following equation^(37)^:
(1)

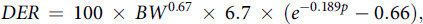




where *P* = BW_*a*_/BW_*e*_, *a* = Actual BW and *e* = expected BW.

During the treatment period, *ad libitum* feeding was mimicked by offering a food dose calculated as three times the cat’s individual DER. The food dose was divided into three meals per d. At each feeding time (08.00 h, 12.00 h and 16.00 h), cats had access to the food for 20 min similar to the feeding patterns of growing kittens^(38)^.

Choline chloride (PetShure 97 % choline chloride; 72.3 % choline) (Balchem) was used for the choline supplement. The choline chloride was diluted into distilled water at a concentration of 500 mg choline chloride/ml distilled water to form a stock solution. Daily choline doses were calculated on an individual basis at 300 mg choline/kg BW^0·75^. This dose was determined from previous research including a pilot study in obese cats^(39)^, and a dose–response study in rats^(40)^, and in growing pigs^(41)^. Choline was supplemented as a top-dress once daily by adding the pipetted amount to a small portion of food (20 g) prior to the 08.00 h feeding time.

### Experimental design

Kittens were acclimated to being handled and restrained by the researchers, as well as acclimated to the housing environment and base diet for 11 weeks between arrival in the colony and gonadectomy. Kittens had blood drawn at the end of the acclimation period (week −1). At week 1, after acclimation, kittens were assigned to two groups; CHOLINE (*n* 8) (mean (±sem) BW of 3·81 kg ± 0·088 kg) and CONTROL (*n* 8) (mean (±sem) BW of 3·75 kg ± 0·088 kg) and blocked into four groups of four containing two CHOLINE and two CONTROL cats for a staggered randomised complete block design. Kittens were anesthetised, and gonadectomy surgeries performed. Anesthesia and surgery procedures were described previously^(34)^. Daily choline supplementation began the day after gonadectomy and continued for 12 weeks for CHOLINE, while CONTROL did not receive the choline supplement throughout the 12-week treatment period. Daily food intake and weekly BW were measured throughout the study^(34)^. Blood samples were collected again from all kittens after the 12-week treatment period (week 12).

### Blood collection and laboratory analyses

Blood collection was completed in the morning after an overnight fast (16 h since last meal) under sedation, to minimise potential stress to the animals, at week −1 and week 12. Sedation was achieved using Butorphanol (Zoetis) (10 mg/ml) at a dose of 0·3 mg/kg BW given intramuscularly alongside intramuscular dexmedetomidine hydrochloride (Dexdomitor, Zoetis) (0·5 mg/ml) at a dose of 0·001 mg/kg BW^(42)^ and reversed with atipamezole (Antisedan, Zoetis) (5 mg/ml) at a dose 0·2 mg/kg BW^(36)^.

Whole blood (ml) was collected by jugular venepuncture with a BD Vacutainer Ultra Touch Push Button 23 G × ¾ and collected in a BD Vacutainer Venous Blood Collection Tubes: Serum Separating Tubes: Hemogard (Becton Dickson) and immediately placed on ice and allowed to clot. Blood was then centrifuged at 2500 rpm for 15 min at 4°C (LegendRT, Kendro Laboratory Products 2002). Serum was pipetted and aliquoted into a 1·5-ml Fisherbrandⓞ Microcentrifuge Tubes (Thermo Fisher Scientific) and stored at −20°C until further analyses.

Serum samples were sent to The Metabolomics Innovation Centre (TMIC) (University of Alberta, Edmonton, AB, Canada) on dry ice for Di-MS and quantitative NMR spectroscopy. For the quantitative NMR spectroscopy, 300 ul were used to analyse water-soluble metabolites such as amino acids, sugars, alcohols, organic acids, amines and SCFA via the Chenomx NMR Suite Professional v. 6.0 (TMIC, University of Alberta, Edmonton, AB, Canada)^(43)^. These metabolites have been classified by their metabolic pathways according to the Human Metabolite Database^(44)^: gluconeogenic amino acids; ketogenic amino acids; augmented amino acid degradation products; glycolysis; lipolysis; TCA cycle; ketogenesis; one-carbon metabolism; purine degradation; alcohols; and ‘other’. Serum (100 ul) was used for Di-MS to analyse metabolites, which have been classified as: amino acids, amino acid derivatives and ammonium compounds; biogenic amines; organic acids and sugars; acyl carnitines; PC; LPC; and SM using a 4000 QTrap mass spectrometer (TMIC, University of Alberta)^(39)^. Total amino acids were calculated as the sum of all presented amino acids. Acyl carnitines were further classified as free (C0), short (C2, C3, C3 OH, C3:1, C4, C4 OH, C4:1, C5, C5 OH, C5 DC, C5 MDC, C5:1, C5:1 DC), medium (C6, C6:1, C7 DC, C8, C9, C10, C10:1, C10:2, C12, C12 DC, C12:1) and long (C14, C14:1 C14:1 OH, C14:2, C14:2 OH, C16, C16 OH, C16:1, C16:1 OH, C16:2, C16:2 OH, C18, C18:1, C18:1 OH, C18:2). Totals for each classification were calculated as the sum of acyl carnitines within each class. Totals for PC diacyls and acyl-alkyls, LPC, hydroxy-sphingomyelins (HSM) and SM were calculated as the sum of each respective group.

### Statistical analyses

Using the standard deviations and differences in means detected in a preliminary feline study performed by the research group that investigated the effect of choline on blood lipid profile, a sample size calculation was performed. Using a power of 0·80 and alpha of 0·05, a total of 10 cats per group are required to detect significant differences using the following equation:

N = (Z*α*(1·96–*α*) + Z*β* (0·84–power))^2^ + (2SD)^2^/(different means)^2^

As this study was able to control for age, sex and breed differences, the sample size was reduced to a total of sixteen cats, with eight cats per treatment.

All Di-MS and NMR spectroscopy data were statistically analysed using Statistical Analysis System (SAS Studio, 3·8, SAS Institute). The residuals for all metabolite data were tested for normality using the Shapiro–Wilk test. If the residuals were not normally distributed, a log-normal distribution was applied to meet the assumptions of ANCOVA. This occurred for: alanine, C7 DC, spermine, creatine, trimethylamine N-oxide and l-leucine. The proc GLIMMIX procedure was used with time as the repeated measure, group as a fixed effect and cat as subject. The covariance structure that resulted in the smallest Akaike information criterion value was used to separate means when there was significance at the fixed effect. The LSMEANS statement was used to calculate the least square means (LSM), and the SLICEDIFF multiple comparison statement was used for comparisons for time and for group. Results are reported as least square means with the standard error of the mean. Effects of group, time and group × time are reported with significance set as *P* < 0·05. A Tukey’s *post hoc* analysis was used to separate the means between and/or within groups as denoted with superscripts (A, B, C and D). Heat maps were developed for statistically significant metabolites from Di-MS and NMR data, respectively, using Metaboanalyst 5.0. Heat maps were created using Euclidean distance measures and clustered using the Ward algorithm.

## Results

Over the study period, all kittens consumed and tolerated the diet and choline supplement with no adverse effects. One kitten (CONTROL) was excluded from the study due to an event unrelated to the study and was therefore not included in statistical analyses.

### Direct infusion MS results

**Fig. 1.**
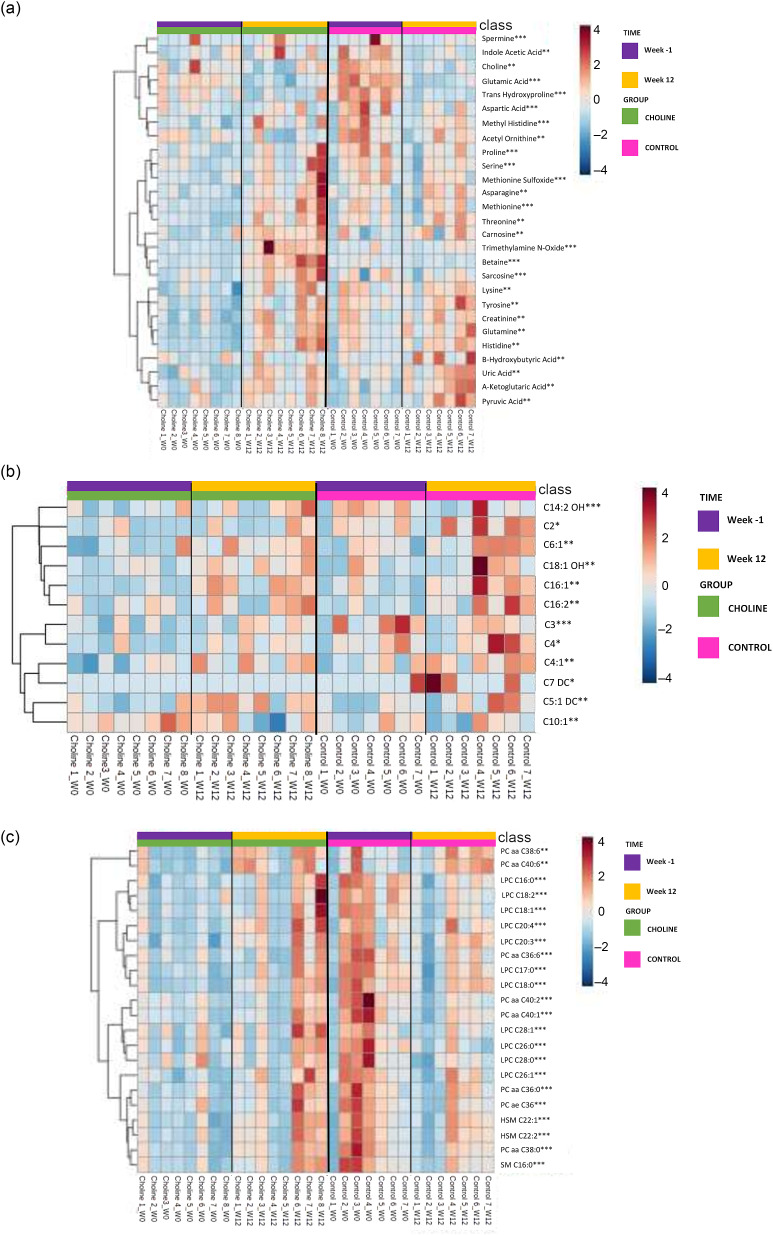
Heat maps with Euclidean distance and Ward clustering of mean serum metabolites analysed by Di-MS in kittens pre-gonadectomy (week −1) and post-gonadectomy (week 12) following supplementation with additional choline at 300 mg/kg BW^0·75^ (CHOLINE, *n* 8) for 12 weeks compared to a control group (CONTROL, *n* 7) and separated by: (a) serum amino acids, amino acid derivatives, ammonium compounds, biogenic amines, and organic sugars and acids; (b) serum acyl carnitines; and (c) serum phosphatidylcholines, lysophosphatidylcholines and sphingomyelins; with a time (*), group (**) or group × time interaction (***) (*P* < 0·05) following a Tukey’s *post hoc* analysis between and within groups.

In Fig. 1, all metabolites from the Di-MS analyses with a significant time, group or group × time effect (*P* < 0·05) are represented in heat maps. Spermine, indole acetic acid, choline, glutamic acid, trans-hydroxyproline, aspartic acid, methylhistidine and acetyl ornithine form a large cluster in which their concentrations appear to decrease over time in CONTROL (Fig. 1(a)). Alternatively, a large cluster including proline, serine, methionine sulfoxide, asparagine, methionine, threonine, carnosine, trimethylamine N-oxide, betaine and sarcosine suggests increased serum concentrations over time in CHOLINE, but not CONTROL. Serum concentrations of lysine, tyrosine, creatinine, glutamine, histidine, b-hydroxybutyric acid, uric acid, a-ketoglutaric acid and pyruvic acid form another large-scale cluster of metabolites that increased in both CHOLINE and CONTROL over time. Acyl carnitines appeared to increase in both groups from week 0 to week 12 (Fig. 1(b)). Serum concentrations of LPC, PC, SM and HSM all appear to follow a similar pattern in which they increased in CHOLINE and decreased in CONTROL (Fig. 1(c)). One notable exception is the small cluster that is formed with PC aa C38:6 and PC aa C40:6 in which serum concentrations appeared to increase in both groups.

### Amino acids, amino acid derivatives and ammonium compounds

Mean serum concentrations of asparagine (*P*_Time_ = 0·03), glutamine (*P*_Time_ = 0·002), glutamic acid (*P*_Time_ < 0·001), histidine (*P*_Time_ = 0·01), lysine (*P*_Time_ = 0·01), methionine (*P*_Time_ = 0·02), threonine (*P*_Time_ = 0·01), tyrosine (*P*_Time_ = 0·04) and choline (*P*_Time_ = 0·04) increased from baseline to 12 weeks post-gonadectomy, regardless of group (Table 1). A group-by-time interaction was observed for aspartic acid (*P*_Group × Time_ = 0·04), glutamic acid (*P*_Group × Time_ = 0·04), methionine (*P*_Group × Time_ = 0·01), proline (*P*_Group × Time_ = 0·04) and serine (*P*_Group × Time_ = 0·04). Serum concentration of aspartic acid decreased for CONTROL, but not for CHOLINE (*P*_Group × Time_ = 0·04). Glutamic acid concentrations were different between groups at week –1, decreased in both groups (*P*_Time_ < 0·001), and were not different between groups at week 12 (*P*_Group × Time_ = 0·04). Total serum methylhistidine concentrations increased significantly in the CHOLINE group only (*P*_Group × Time_ = 0·03). Betaine concentrations appeared to decrease in the CONTROL group and significantly increased in the CHOLINE group after 12 weeks (*P*_Group × Time_ < 0·001). No effect of group or time was noted for serum concentrations of alanine, arginine, citrulline, glycine, isoleucine, leucine, ornithine, phenylalanine, tryptophan, and valine, or for total amino acids, and creatine.

**Table 1.** Mean serum concentrations of amino acids, amino acid derivates and ammonium compounds analysed by Di-MS in kittens pre-gonadectomy (week −1) and post-gonadectomy (week 12) following supplementation with additional choline at 300 mg/kg BW^0·75^ (CHOLINE, *n* 8) for 12 weeks compared to a control group (CONTROL, *n* 7) fed only the base diet (3310 mg choline/kg DM)

		Week	CHOLINE (*n* 8)		CONTROL (*n* 7)				
Metabolites (µM)			LSM	sem	LSM	sem	*P*_Group_	*P*_Time_	*P*_Group × Time_
Amino acids	Alanine	–1	447·35	41·91	451·79	39·18	0·48	0·22	0·37
		12	534·68		465·08				
	Arginine	–1	181·72	15·85	225·18	17·04	0·72	0·58	0·06
		12	229·97		197·89				
	Asparagine	–1	45·69^A^	5·56	50·63^A^	5·96	0·89	0·03	0·32
		12	65·70^B^		59·00^B^				
	Aspartic acid	–1	58·04	5·96	66·77^A^	6·39	0·50	0·11	0·04
		12	61·05		43·10^B^				
	Citrulline	–1	24·96	2·28	26·52	2·45	0·99	0·15	0·50
		12	30·01		28·45				
	Glutamine	–1	609·9^A^	56·97	756·26^A^	61·53	0·36	0·002	0·18
		12	895·56^B^		884·97^B^				
	Glutamic acid	–1	159·16^Ac^	6·99	185·53^Ad^	7·53	0·27	< 0·001	0·04
		12	119·25^B^		108·66^B^				
	Glycine	–1	450·00	24·91	483·00	26·73	0·59	0·06	0·08
		12	447·25		383·86				
	Histidine	–1	96·48^A^	8·06	112·50^A^	8·68	0·85	0·01	0·09
		12	142·45^B^		130·25^B^				
	Isoleucine	–1	60·00	5·61	65·63	6·03	0·64	0·33	0·15
		12	74·38		62·63				
	Leucine	–1	131·16	14·55	152·61	15·68	0·82	0·12	0·09
		12	180·20		150·56				
	Lysine	–1	169·94^A^	14·26	193·21^A^	15·34	0·85	0·01	0·08
		12	236·44^B^		206·64^B^				
	Methionine	–1	48·13^A^	4·70	50·64	5·05	0·06	0·02	0·01
		12	74·61^Bc^		49·69^d^				
	Ornithine	–1	25·79	2·95	28·54	3·17	0·82	0·60	0·24
		12	30·93		26·51				
	Phenylalanine	–1	75·19	7·04	84·43	7·55	0·96	0·05	0·19
		12	99·85		89·81				
	Proline	–1	140·38	12·68	155·90	13·67	0·39	0·95	0·04
		12	170·21^c^		127·72^d^				
	Serine	–1	147·19^A^	13·15	155·22	14·12	0·17	0·39	0·04
		12	189·06^Bc^		136·89^d^				
	Threonine	–1	119·77^A^	14·62	134·95^A^	15·71	0·81	0·01	0·21
		12	183·33^B^		159·93^B^				
	Tyrosine	–1	46·64^A^	6·23	59·73^A^	6·72	0·29	0·04	0·46
		12	65·09^B^		68·73^B^				
	Tryptophan	–1	85·82	7·10	102·15	7·61	0·60	0·35	0·16
		12	107·41		97·45				
	Valine	–1	177·41	15·88	198·24	17·06	0·97	0·07	0·19
		12	230·04		206·81				
	Total amino acids	–1	3074·25	242·74	3604·32	262·21	0·68	0·12	0·11
		12	3868·49		3594·05				
Amino acid derivatives	Total methylhistidine	–1	33·23^A^	2·61	37·08	2·81	0·42	0·25	0·03
		12	42·77^Bc^		33·75^d^				
	Creatine	–1	12·85	1·55	14·05	1·34	0·54	0·49	0·18
		12	16·08		13·02				
	Betaine	–1	166·75^A^	37·30	210·29	40·27	0·005	0·001	<0·001
		12	515·12^Bc^		155·86^d^				
Ammonium compounds	Choline	–1	19·61	1·56	21·22^A^	1·67	0·98	0·04	0·42
		12	16·28		14·59^B^				

LSM, least square means.

Rows or columns with no superscript indicates no significant differences for the measured parameter.

Down a column, different upper-case superscript letters (A, B) denote significant differences (*P* < 0·05) following a Tukey’s *post hoc* analysis within group (CHOLINE or CONTROL) over time (week −1 to week 12).

Across a row, different lower-case letters (c, d) denote significant differences (*P* < 0·05) following a Tukey’s *post hoc* analysis between groups (CHOLINE and CONTROL) at one time point (week −1 or week 12).

### Biogenic amines

Mean serum biogenic amine concentrations are summarised in Table 2. An effect of time was observed, regardless of group, for acetyl ornithine (*P*_Time_ = 0·02), carnosine (*P*_Time_ < 0·001) and creatinine (*P*_Time_ = 0·01). Methionine sulfoxide increased from week –1 to week 12 for CHOLINE but decreased for CONTROL (*P*_Group × Time_ = 0·03). Serum concentrations of trans-hydroxyproline decreased significantly in both groups (*P*_Time_ < 0·001), with a larger decrease in the CONTROL group (*P*_Group × Time_ = 0·008). Serum concentration of sarcosine increased significantly for CHOLINE but not for CONTROL (*P*_Group × Time_ = 0·007). Alternatively, spermine decreased for CONTROL but not for CHOLINE (*P*_Group × Time_ = 0·03). Trimethylamine N-oxide serum concentrations increased significantly in both groups (*P*_Time_ < 0·001), with a larger increase in the CONTROL group (*P*_Group × Time_ = 0·002). There was no significant effect of group or time for asymmetric dimethylarginine, *α*-aminoadipic acid, kynurenine, putrescine, serotonin and spermidine.

**Table 2.** Mean serum concentrations of biogenic amines analysed by Di-MS in kittens pre-gonadectomy (week −1) and post-gonadectomy (week 12) after supplementation with additional choline at 300 mg/kg BW^0·75^ (CHOLINE, *n* 8) for 12 weeks compared to a control group (CONTROL, *n* 7) fed only the base diet (3310 mg choline/kg DM)

			CHOLINE (*n* 8)		CONTROL (*n* 7)				
	Metabolites (µM)	Week	LSM	sem	LSM	sem	*P*_Group_	*P*_Time_	*P*_Group × Time_
Biogenic amines	Acetyl ornithine	–1	5·21^A^	0·42	5·49^A^	0·45	0·47	0·02	0·89
		12	3·97^B^		4·37^B^				
	Asymmetric dimethylarginine	–1	4·39	0·32	5·07	0·34	0·60	0·83	0·15
		12	4·93		4·66				
	*α*-Amino adipic acid	–1	1·59	0·009	1·58	0·10	0·51	0·21	0·45
		12	1·64		1·79				
	Carnosine	–1	27·28^A^	3·57	27·38^A^	3·81	0·29	<0·001	0·28
		12	46·69^B^		41·37^B^				
	Creatinine	–1	115·44^A^	11·18	135·77^A^	11·99	0·83	0·01	0·21
		12	173·45^B^		157·48^B^				
	Kynurenine	–1	12·05	1·13	16·09	1·22	0·05	0·45	0·15
		12	13·21		12·59				
	Methionine sulfoxide	–1	7·51^A^	0·80	7·58	0·86	0·06	0·31	0·03
		12	10·33^Bc^		6·46^d^				
	Trans-hydroxyproline	–1	75·16^A^	5·61	85·09^A^	6·05	0·34	<0·001	0·008
		12	52·27^Bc^		33·33^Bd^				
	Putrescine	–1	1·21	0·12	1·62	0·12	0·08	0·10	0·08
		12	1·26		1·03				
	Sarcosine	–1	4·73^A^	0·59	5·53	0·70	0·08	0·08	0·007
		12	7·93^Bc^		4·67^d^				
	Serotonin	–1	30·95	4·97	41·20	5·32	0·17	0·08	0·44
		12	23·72		23·72				
	Spermidine	–1	0·30	0·04	0·40	0·05	0·67	0·94	0·11
		12	0·37		0·32				
	Spermine	–1	0·11	0·009	0·11^A^	0·009	0·16	0·43	0·03
		12	0·12^c^		0·09^Bd^				
	Taurine	–1	129·15	12·65	137·42	13·53	0·58	0·26	0·43
		12	123·29		105·07				
	Trimethylamine-N-oxide	–1	4·91^A^	3·45	6·03	1·33	0·04	<0·001	0·002
		12	6·86^Ac^		7·81^d^				

LSM, least square means.

Rows or columns with no superscript indicates no significant differences for the measured parameter.

Down a column, different upper-case superscript letters (A, B) denote significant differences (*P* < 0·05) following a Tukey’s *post hoc* analysis within group (CHOLINE or CONTROL) over time (week −1 to week 12).

Across a row, different lower-case letters (c, d) denote significant differences (*P* < 0·05) following a Tukey’s *post hoc* analysis between groups (CHOLINE and CONTROL) at one time point (week −1 or week 12).

### Organic acids and sugars

Mean serum concentrations of organic acids and sugars are summarised in Table 3. CONTROL kittens experienced a significant increase in serum concentrations of *β*-hydroxybutyric acid (*P*_Time_ = 0·04) but not those in the CHOLINE group. Both groups had increased concentrations of serum *α*-ketaglutaric acid (*P*_Time_ < 0·001), pyruvic acid (*P*_Time_ = 0·002) and uric acid (*P*_Time_ = 0·001) but no differences in concentrations between groups at either time point. A significant decrease in serum concentration of indole acetic acid was observed only for CONTROL from week –1 to week 12 (*P*_Group × Time_ = 0·007). Post-gonadectomy kittens had no significant differences between or within groups for serum concentrations of lactic acid, citric acid, butyric acid, propionic acid, hydroxy hippuric acid, succinic acid, fumaric acid, isobutyric acid, hippuric acid, methylmalonic acid and glucose.

**Table 3.** Mean serum concentrations of organic acids and sugars analysed by Di-MS in kittens pre-gonadectomy (week −1) and post-gonadectomy (week 12) after supplementation with additional choline at 300 mg/kg BW^0·75^ (CHOLINE, *n* 8) for 12 weeks compared with a control group (CONTROL, *n* 7) fed only the base diet (3310 mg choline/kg DM)

			CHOLINE (*n* 8)		CONTROL (*n* 7)				
Metabolites (µM)		Week	LSM	sem	LSM	sem	*P*_Group_	*P*_Time_	*P*_Group × Time_
Organic acids	Lactic acid	–1	1415·27	171·55	1700·98	183·67	0·05	0·61	0·99
		12	1298·52		1588·00				
	*β*-Hydroxybutyric acid	–1	57·29	13·61	56·74^A^	14·55	0·07	0·04	0·06
		12	59·92^c^		117·38^Bd^				
	*α*-Ketoglutaric acid	–1	8·08^A^	0·99	7·71^A^	1·06	0·39	<0·001	0·23
		12	11·53^B^		13·74^B^				
	Citric acid	–1	235·76	18·99	266·13	20·38	0·12	0·06	0·77
		12	269·76		311·42				
	Butyric acid	–1	0·53	0·07	0·513	0·07	0·14	0·21	0·27
		12	0·36		0·55				
	Propionic acid	–1	1·42	0·25	1·73	0·025	0·47	0·19	0·58
		12	1·18		1·15				
	p-Hydroxyhippuric acid	–1	0·087	0·01	0·11	0·01	0·54	0·09	0·47
		12	0·071		0·07				
	Succinic acid	–1	1·50	0·14	1·62	0·15	0·61	0·57	0·74
		12	1·66		1·66				
	Fumaric acid	–1	1·10	0·15	1·20	0·16	0·43	0·38	0·85
		12	1·21		1·36				
	Pyruvic acid	–1	10·18^A^	2·49	9·42^A^	2·66	0·36	0·002	0·23
		12	16·77^B^		22·42^B^				
	Isobutyric acid	–1	2·64	0·38	2·90	0·41	0·89	0·10	0·60
		12	2·17		2·02				
	Hippuric acid	–1	2·33	0·62	3·64	0·67	0·89	0·88	0·08
		12	3·36		2·41				
	Methylmalonic acid	–1	0·37	0·04	0·36	0·05	0·35	0·21	0·28
		12	0·26		0·35				
	Indole acetic acid	–1	2·22	0·38	2·72^A^	0·41	0·15	0·21	0·007
		12	2·92^c^		1·04^Bd^				
	Uric acid	–1	10·48^A^	0·99	11·58^A^	1·06	0·53	0·001	0·69
		12	15·02^B^		15·32^B^				
Sugars	Glucose	–1	15 427	1543·92	17 332	1655·75	0·96	0·75	0·27
		12	17 741		16 030				

LSM, least square means.

Rows or columns with no superscript indicates no significant differences for the measured parameter.

Down a column, different upper-case superscript letters (A, B) denote significant differences (*P* < 0·05) following a Tukey’s *post hoc* analysis within group (CHOLINE or CONTROL) over time (week −1 to week 12).

Across a row, different lower-case letters (c, d) denote significant differences (*P* < 0·05) following a Tukey’s *post hoc* analysis between groups (CHOLINE and CONTROL) at one time point (week −1 or week 12).

### Acyl carnitines

Mean serum concentrations of acyl carnitines are summarised in Table 4. Serum concentrations of C4 and C7 DC were lower in CHOLINE compared with CONTROL (*P*_Group_ = 0·04 and 0·03, respectively). Regardless of group, serum concentrations of C16:1 (*P*_Time_ = 0·001), C16:2 (*P*_Time_ = 0·04), C18:1 OH (*P*_Time_ = 0·04), C5:1 DC (*P*_Time_ = 0·004), C4:1 (*P*_Time_ = 0·01) and C6:1 (*P*_Time_ = 0·01) increased in both groups 12 weeks post-gonadectomy. Serum concentrations of C10:1 decreased in both groups 12 weeks post-gonadectomy (*P*_Time_ = 0·02). An interaction of group and time was observed in serum concentrations of C3 (*P*_Group × Time_ = 0·04); however, a *post hoc* adjustment showed no significant differences between and within groups. Total short-chain acyl carnitines increased over the 12 weeks regardless of group (*P*_Time_ = 0·02). Similarly, total long-chain acyl carnitines increased over the 12 weeks, regardless of group (*P*_Time_ = 0·04). For all acyl carnitines, there appeared to be an increase from week −1 to week 12 in both groups (*P*_Time_ = 0·03). No significant effect of time or group was observed for C0, C2, C5, C5:1, C12, C14; C14:1, C14:1 OH, C14:2, C14:2 OH, C16, C16:1 OH, C18, C10, C10:2, C12 DC, C12:1, C3 OH, C3:1, C4 OH, C5 DC, C5 MDC, C5 OH, C6, C8, C9, C16:2 OH and C16 OH.

**Table 4.** Mean serum concentrations of acyl carnitines analysed by Di-MS in kittens pre-gonadectomy (week −1) and post-gonadectomy (week 12) after supplementation with additional choline at 300 mg/kg BW^0·75^ (CHOLINE, *n* 8) for 12 weeks compared with a control group (CONTROL, *n* 7) fed only the base diet (3310 mg choline/kg DM)

			CHOLINE (*n* 8)		CONTROL (*n* 7)				
Metabolites (µM)		Week	LSM	sem	LSM	sem	*P*_Group_	*P*_Time_	*P*_Group × Time_
Free	C0	–1	5·96	0·48	6·64	0·52	0·77	0·06	0·32
		12	7·54		7·17				
Short	C2	–1	0·70	0·08	0·83	0·08	0·05	0·10	0·59
		12	0·8		1·02				
	C3	–1	0·065^c^	0·01	0·089^d^	0·01	0·10	0·06	0·04
		12	0·07		0·073				
	C3 OH	–1	0·019	0·001	0·019	0·001	0·80	0·97	0·94
		12	0·019		0·02				
	C3:1	–1	0·01	0·001	0·012	0·001	0·48	0·26	0·56
		12	0·0096		0·0098				
	C4	–1	0·038	0·01	0·053	0·01	0·04	0·10	0·96
		12	0·049		0·065				
	C4 OH	–1	0·023	0·001	0·024	0·001	0·50	0·21	0·80
		12	0·021		0·022				
	C4:1	–1	0·016^A^	0·001	0·018	0·001	0·35	0·01	0·76
		12	0·02^B^		0·02				
	C5	–1	0·052	0·01	0·081	0·01	0·08	0·16	0·30
		12	0·075		0·085				
	C5 OH	–1	0·023	0·002	0·025	0·002	0·26	0·07	0·89
		12	0·026		0·029				
	C5 DC	–1	0·012	0·001	0·01	0·001	0·62	0·93	0·23
		12	0·012		0·012				
	C5 MDC	–1	0·017	0·001	0·015	0·001	0·28	0·96	0·49
		12	0·017		0·016				
	C5:1	–1	0·018	0·002	0·022	0·002	0·21	0·62	0·62
		12	0·02		0·022				
	C5:1 DC	–1	0·012^A^	0·001	0·012^A^	0·001	0·65	0·004	0·79
		12	0·016^B^		0·015^B^				
Medium	C6	–1	0·046	0·003	0·043	0·003	0·92	0·48	0·36
		12	0·045		0·047				
	C6:1	–1	0·030^A^	0·002	0·031^A^	0·002	0·47	0·01	0·91
		12	0·036^B^		0·038^B^				
	C7 DC	–1	0·0084	0·003	0·012	0·012	0·03	0·14	0·19
		12	0·0089^c^		0·033^d^				
	C8	–1	0·014	0·001	0·017	0·001	0·18	0·13	0·13
		12	0·018		0·017				
	C9	–1	0·0072	0·001	0·0081	0·001	0·46	0·25	0·63
		12	0·0085		0·0087				
	C10	–1	0·04	0·002	0·04	0·002	0·92	0·20	0·91
		12	0·037		0·037				
	C10:1	–1	0·089^A^	0·005	0·08^A^	0·005	0·18	0·02	0·78
		12	0·077^B^		0·07^B^				
	C10:2	–1	0·013	0·001	0·013	0·001	0·99	0·63	0·88
		12	0·014		0·014				
	C12	–1	0·032	0·002	0·033	0·002	0·70	0·13	0·97
		12	0·029		0·030				
	C12 DC	–1	0·017	0·001	0·015	0·001	0·16	0·58	0·16
		12	0·016		0·016				
	C12:1	–1	0·052	0·003	0·049	0·003	0·43	0·33	0·98
		12	0·049		0·046				
Long	C14	–1	0·021	0·002	0·023	0·002	0·44	0·27	0·78
		12	0·024		0·025				
	C14:1	–1	0·069	0·007	0·083	0·008	0·99	0·56	0·07
		12	0·087		0·073				
	C14:1 OH	–1	0·011	0·001	0·013	0·001	0·33	0·26	0·56
		12	0·013		0·014				
	C14:2	–1	0·012	0·001	0·012	0·001	0·66	0·09	0·63
		12	0·014		0·015				
	C14:2 OH	–1	0·014	0·001	0·016	0·001	0·85	0·50	0·05
		12	0·016		0·014				
	C16	–1	0·10	0·01	0·12	0·015	0·94	0·13	0·25
		12	0·15		0·13				
	C16 OH	–1	0·0082	0·001	0·0083	0·001	0·35	0·26	0·43
		12	0·0086		0·01				
	C16:1	–1	0·035^A^	0·003	0·038^A^	0·003	0·68	0·001	0·57
		12	0·052^B^		0·051^B^				
	C16:1 OH	–1	0·013	0·001	0·015	0·001	0·60	0·09	0·61
		12	0·016		0·016				
	C16:2	–1	0·01^A^	0·001	0·0093^A^	0·001	0·81	0·04	0·58
		12	0·012^B^		0·013^B^				
	C16:2 OH	–1	0·0084	0·006	0·0080	0·001	0·37	0·77	0·89
		12	0·0087		0·0081				
	C18	–1	0·072	0·009	0·089	0·01	0·23	0·15	0·56
		12	0·096		0·099				
	C18:1	–1	0·087	0·02	0·104	0·02	0·94	0·10	0·33
		12	0·14		0·12				
	C18:1 OH	–1	0·0097^A^	0·001	0·0098^A^	0·001	0·52	0·04	0·42
		12	0·012^B^		0·011^B^				
	C18:2	–1	0·034	0·005	0·038	0·006	0·94	0·09	0·53
		12	0·049		0·045				
Totals	Total short-chain AC	–1	1·043^A^	0·09	1·116^A^	0·09	0·17	0·02	0·44
		12	1·194^B^		1·402^B^				
	Total medium-chain AC	–1	0·346	0·02	0·352	0·02	0·05	0·33	0·16
		12	0·337^c^		0·403^d^				
	Total long-chain AC	–1	0·503^A^	0·06	0·552^A^	0·07	0·74	0·05	0·68
		12	0·686^B^		0·676^B^				
	Total AC	–1	7·838^A^	0·60	8·677^A^	0·64	0·60	0·03	0·45
		12	9·747^B^		9·659^B^				

LSM, least square means.

Rows or columns with no superscript indicates no significant differences for the measured parameter.

Down a column, different upper-case superscript letters (A, B) denote significant differences (*P* < 0·05) following a Tukey’s *post hoc* analysis within group (CHOLINE or CONTROL) over time (week −1 to week 12).

Across a row, different lower-case letters (c, d) denote significant differences (*P* < 0·05) following a Tukey’s *post hoc* analysis between groups (CHOLINE and CONTROL) at one time point (week −1 or week 12).

Short = C2 + C3 + C3 OH + C3:1 + C4 + C4 OH + C4:1 + C5 + C5 OH + C5 DC + C5 MDC + C5:1 + C5:1 DC.

Medium = C6 + C6:1 + C7 DC + C8 + C9 + C10 + C10:1 + C10:2 + C12 + C12 DC + C12:1.

Long = C14 + C14:1 + C14:1OH + C14:2 + C14:2OH + C16 + C16OH + C16:1 + C16:1OH + C16:2 + C16:2OH + C18 + C18:1 + C18:1 OH + C18.

### Phosphatidylcholines

Mean serum PC diacyl (aa) and PC acyl-alkyl (ae) concentrations for the CHOLINE and CONTROL groups at week −1 and week 12 are summarised in Table 5. An effect of time was observed in serum concentrations of PC aa C38:6 (*P*_Time_ = 0·01) and PC aa C40:6 (*P*_Time_ = 0·003) regardless of group. Serum concentrations of PC aa C36:6 decreased significantly for CONTROL with no changes in CHOLINE (*P*_Group × Time_ = 0·03). CHOLINE experienced a significant increase in serum concentrations of PC aa C38:0 after 12 weeks of choline supplementation, whereas there were no changes for CONTROL (*P*_Group × Time_ = 0·02). Total PC aa increased over 12 weeks in both groups regardless of group (*P*_Time_ = 0·009). Total PC ae increased for CHOLINE but not for CONTROL after 12 weeks (*P*_Group × Time_ = 0·04). After 12 weeks, no changes were found in serum concentrations of PC aa C40:2, PC ae C36 and PC aa C40:1 for CHOLINE; however, there was a significant decrease for CONTROL (*P*_Group × Time_ = 0·020, *P*_Group × Time_ = 0·04, *P*_Group × Time_ = 0·01, respectively). Serum concentrations of PC aa C32:2, PC aa C36:0 and PC ae C40:6 were not affected by group or time.

**Table 5.** Mean serum concentrations of phosphatidylcholines (PC) analysed by Di-MS in kittens pre-gonadectomy (week −1) and post-gonadectomy (week 12) after supplementation with additional choline at 300 mg/kg BW^0·75^ (CHOLINE, *n* 8) for 12 weeks compared to a control group (CONTROL, *n* 7) fed only the base diet (3310 mg choline/kg DM)

			CHOLINE (*n* 8)		CONTROL (*n* 7)				
Metabolites (µM)		Week	LSM	sem	LSM	sem	*P*_Group_	*P*_Time_	*P*_Group × Time_
PC aa	PC aa C32:2	–1	3·76	0·40	4·30	0·43	0·75	0·67	0·11
		12	4·62		3·79				
	PC aa C36:0	–1	20·23	2·23	24·09	2·39	0·71	0·68	0·05
		12	26·04		20·15				
	PC aa C36:6	–1	1·27	0·13	1·45^A^	0·14	0·41	0·29	0·03
		12	1·44^c^		0·99^Bd^				
	PC aa C38:0	–1	3·93	0·36	4·80	0·39	0·46	0·96	0·02
		12	5·07^c^		3·70^d^				
	PC aa C38:6	–1	58·84^A^	7·49	65·97^A^	8·02	0·71	0·01	0·26
		12	93·27^B^		80·67^B^				
	PC aa C40:2	–1	1·87	0·18	2·32^A^	0·20	0·40	0·08	0·02
		12	2·03^c^		1·30^Bd^				
	PC aa C40:6	–1	67·90^A^	9·65	75·02^A^	10·33	0·95	0·003	0·44
		12	112·35^B^		103·86^B^				
PC ae	PC ae C36	–1	3·20	0·32	3·68	0·34	0·52	0·63	0·04
		12	3·77		2·79				
	PC ae C40:6	–1	4·33	0·45	5·04	0·48	0·64	0·13	0·05
		12	6·01		4·81				
	PC aa C40:1	–1	0·79	0·08	0·95^A^	0·08	0·47	0·41	0·01
		12	0·95^c^		0·66^Bd^				
Totals	PC aa	–1	158·19^A^	19·70	179·33^A^	21·15	0·84	0·009	0·22
		12	245·39^B^		215·55^B^				
	PC ae	–1	6·748^A^	0·84	9·323	0·87	0·46	0·36	0·04
		12	9·49^B^		8·211				

LSM, least square means.

Rows or columns with no superscript indicates no significant differences for the measured parameter.

Down a column, different upper-case superscript letters (A, B) denote significant differences (*P* < 0·05) following a Tukey’s *post hoc* analysis within group (CHOLINE or CONTROL) over time (week −1 to week 12).

Across a row, different lower-case letters (c, d) denote significant differences (*P* < 0·05) following a Tukey’s *post hoc* analysis between groups (CHOLINE and CONTROL) at one time point (week −1 or week 12).

### Lysophosphatidylcholines

Mean serum concentrations of LPC are summarised in Table 6. There was a significant increase in serum LPC C20:4, LPC C20:3, LPC C28:1 and LPC C26:1 for CHOLINE after 12 weeks of additional choline supplementation in post-gonadectomy kittens (*P*_Group × Time_ = 0·02, *P*_Group × Time_ = 0·04. *P*_Group × Time_ = 0·03 and *P*_Group × Time_ = 0·03, respectively). Serum concentrations of LPC 18:2 increased significantly for CHOLINE and significantly decreased in CONTROL (*P*_Group × Time_ = 0·01). A group × time interaction was noted for LPC C16:0 and LPC C18:1 (*P*_Group × Time_ = 0·02 and *P*_Group × Time_ = 0·01, respectively). While there was no significance within groups for LPC C16:0 and LPC C18:1, the CHOLINE group experienced a slight increase in serum concentrations, while the CONTROL group had a slight decrease from week −1 to week 12, leading to a significant difference between groups at week 12. There was an increase in serum concentrations of LPC C17:0 for CHOLINE and a decrease in serum concentration for CONTROL (*P*_Group × Time_ = 0·02). Serum concentration of LPC 26:0 significantly decreased in the CONTROL group from week −1 to week 12 with no changes for CHOLINE (*P*_Group × Time_ = 0·004). Total LPC appeared to have a group × time interaction; however, a *post hoc* adjustment showed that while CHOLINE increased and CONTROL decreased in total LPC concentrations, these were only trends and similarly, the *post hoc* adjustment showed that at week 12, CHOLINE tended to be greater than CONTROL but was not significant (*P*_Group × Time_ = 0·02). No significant differences were found in LPC C14:0, C16:1, C18:0 and C24:0.

**Table 6.** Mean serum concentrations of lysophosphatidylcholines (LPC) analysed by Di-MS in kittens pre-gonadectomy (week −1) and post-gonadectomy (week 12) after supplementation with additional choline at 300 mg/kg BW^0·75^ (CHOLINE, *n* 8) for 12 weeks compared with a control group (CONTROL, *n* 7) fed only the base diet (3310 mg choline/kg DM)

			CHOLINE (*n* 8)		CONTROL (*n* 7)				
	Metabolites (µM)	Week	LSM	sem	LSM	sem	*P*_Group_	*P*_Time_	*P*_Group × Time_
LPC	LPC C14:0	–1	1·62	0·14	1·77	0·16	0·61	0·61	0·10
		12	1·79		1·45				
	LPC C16:1	–1	2·94	0·36	3·26	0·39	0·34	0·89	0·06
		12	3·63		2·48				
	LPC C16:0	–1	51·90	5·82	64·19	6·28	0·71	0·75	0·02
		12	65·14		47·29				
	LPC C17:0	–1	2·33	0·27	3·01	0·29	0·93	0·91	0·02
		12	2·95		2·33				
	LPC C18:2	–1	33·51	4·31	36·06	4·65	0·10	0·89	0·01
		12	44·97^c^		23·4^d^				
	LPC C18:1	–1	22·20	2·87	26·06	3·10	0·29	0·84	0·01
		12	29·52^c^		17·63^d^				
	LPC C18:0	–1	61·86	6·73	82·48	7·25	0·62	0·55	0·05
		12	83·83		70·15				
	LPC C20:4	–1	5·78^A^	0·68	6·35	0·73	0·17	0·10	0·02
		12	8·67^Bc^		5·81^d^				
	LPC C20:3	–1	2·55	0·20	2·93	0·22	0·82	0·19	0·04
		12	3·26^c^		2·77^d^				
	LPC C24:0	–1	0·23	0·03	0·26	0·03	0·44	0·13	0·11
		12	0·33		0·26				
	LPC C26:1	–1	0·06	0·01	0·085^A^	0·01	0·86	0·85	0·03
		12	0·09^c^		0·06^Bd^				
	LPC C26:0	–1	0·10	0·01	0·12^A^	0·01	0·06	0·20	0·004
		12	0·13^c^		0·069^Bd^				
	LPC C28:1	–1	0·18	0·02	0·20	0·02	0·10	0·63	0·03
		12	0·24^c^		0·16^d^				
	LPC C28:0	–1	0·17	0·02	0·19	0·02	0·13	0·13	0·04
		12	0·18		0·12				
Totals	LPC	–1	184·17^A^	21·40	228·39^A^	23·10	0·67	0·88	0·02
		12	243·45^B^		175·41^B^				

LSM, least square means.

Rows or columns with no superscript indicates no significant differences for the measured parameter.

Down a column, different upper-case superscript letters (A, B) denote significant differences (*P* < 0·05) following a Tukey’s *post hoc* analysis within group (CHOLINE or CONTROL) over time (week −1 to week 12).

Across a row, different lower-case letters (c, d) denote significant differences (*P* < 0·05) following a Tukey’s *post hoc* analysis between groups (CHOLINE and CONTROL) at one time point (week −1 or week 12).

### Sphingomyelins

Mean HSM and SM concentrations of CHOLINE and CONTROL groups at week −1 and week 12 are summarised in Table 7. Serum concentrations of HSM C22:1 and C22:2 significantly increased after 12 weeks for CHOLINE, with no change for CONTROL, resulting in a significant difference between groups at week 12 (*P*_Group × Time_ = 0·04). Similarly, a significant increase in serum concentrations of SM C16:0 was observed for CHOLINE which resulted in a significant difference between groups at week 12 (*P*_Group × Time_ = 0·02). Total HSM increased for CHOLINE after 12 weeks but not for CONTROL (*P*_Group × Time_ = 0·04). There were no significant effects of time or group on HSM C14:1, C16:1 and C24:1. Similarly, no effects of time or group were observed in serum concentrations of SM C16:1, C18:0, C18:1, C20:2 or in total SM.

**Table 7.** Mean serum concentrations of hydroxysphingomyelins (HSM) and sphingomyelins (SM) analysed by Di-MS in kittens pre-gonadectomy (week −1) and post-gonadectomy (week 12) after supplementation with additional choline at 300 mg/kg BW^0·75^ (CHOLINE, *n* 8) for 12 weeks compared with a control group (CONTROL, *n* 7) fed only the base diet (3310 mg choline/kg DM)

			CHOLINE (*n* 8)		CONTROL (*n* 7)				
Metabolites (µM)		Week	LSM	sem	LSM	sem	*P*_Group_	*P*_Time_	*P*_Group × Time_
HSM	HSM C14:1	–1	7·75	0·84	8·83	0·90	0·51	0·21	0·06
		12	10·60		8·22				
	HSM C16:1	–1	3·28	0·36	3·93	0·38	0·93	0·35	0·17
		12	4·32		3·72				
	HSM C22:1	–1	14·84^A^	1·44	17·39	1·55	0·67	0·26	0·04
		12	19·81^B^		15·79				
	HSM C22:2	–1	7·49	0·70	8·57	0·75	0·55	0·54	0·04
		12	9·49		7·44				
	HSM C24:1	–1	2·37	0·22	2·95	0·24	0·87	0·51	0·06
		12	3·10		2·59				
SM	SM C16:0	–1	124·17	1·66	148·75	12·54	0·67	0·95	0·02
		12	153·93		117·42				
	SM C16:1	–1	4·57	0·44	5·31	0·47	0·48	0·99	0·07
		12	5·60		4·29				
	SM C18:0	–1	21·43	2·31	25·07	2·47	0·81	0·39	0·16
		12	28·05		23·43				
	SM C18:1	–1	4·70	0·50	5·48	0·54	0·76	0·74	0·17
		12	5·82		4·78				
	SM C20:2	–1	0·32	0·04	0·34	0·04	0·32	0·40	0·09
		12	0·42		0·30				
Totals	HSM	–1	35·68^A^	3·53	41·72	3·79	0·68	0·29	0·04
		12	47·27^B^		37·81				
	SM	–1	155·14	14·36	185·00	15·41	0·61	0·91	0·05
		12	193·77		150·28				

LSM, least square means.

Rows or columns with no superscript indicates no significant differences for the measured parameter.

Down a column, different upper-case superscript letters (A, B) denote significant differences (*P* < 0·05) following a Tukey’s *post hoc* analysis within group (CHOLINE or CONTROL) over time (week −1 to week 12).

Across a row, different lower-case letters (c, d) denote significant differences (*P* < 0·05) following a Tukey’s *post hoc* analysis between groups (CHOLINE and CONTROL) at one time point (week −1 or week 12).

### NMR results

**Fig. 2.**
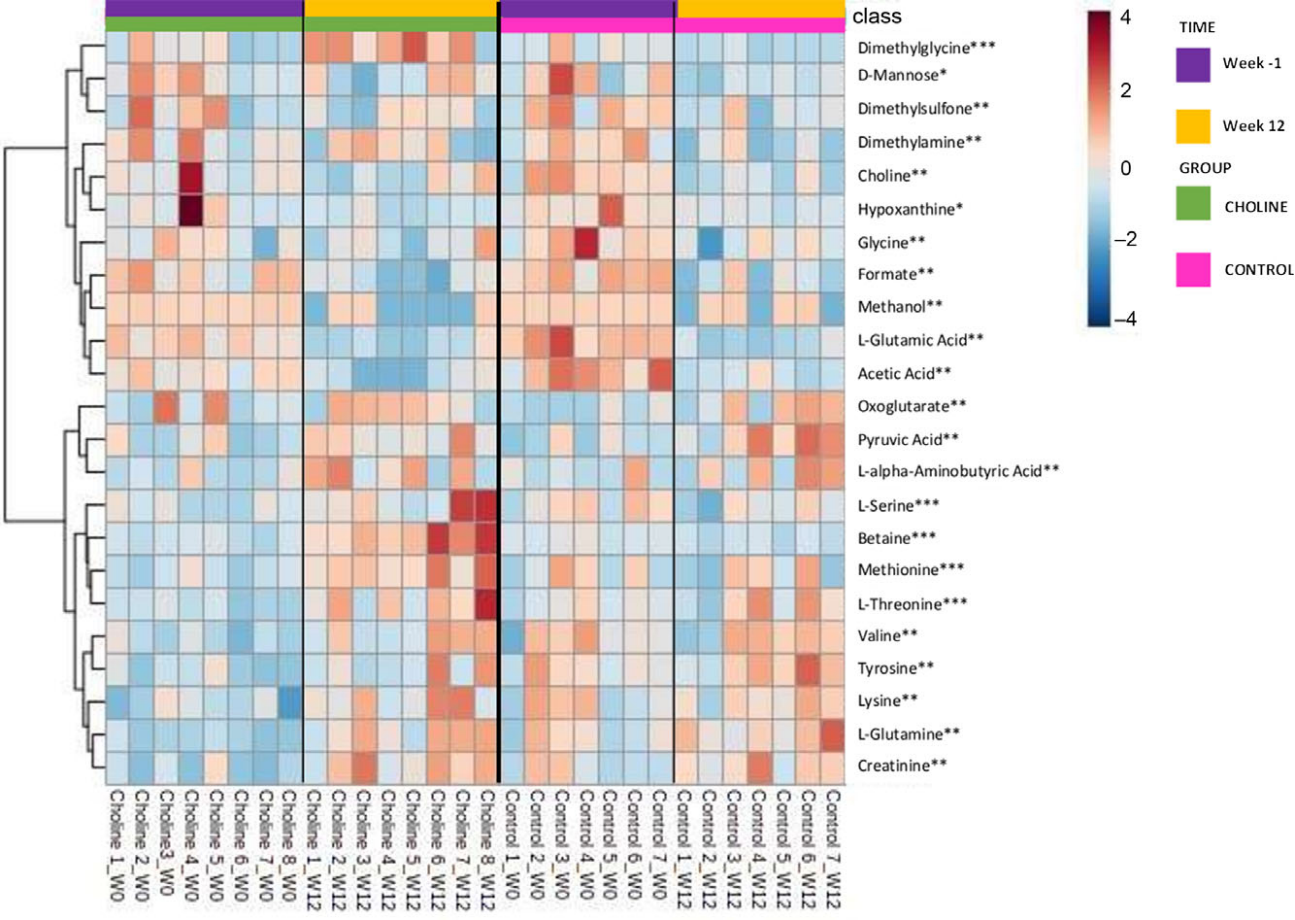
Heat map with Euclidean distance and Ward clustering of mean serum metabolites analysed by quantitative NMR spectrometry in kittens pre-gonadectomy (week −1) and post-gonadectomy (week 12) following supplementation with additional choline at 300 mg/kg BW 0·75 (CHOLINE, *n* 8) for 12 weeks compared to a control group (CONTROL, *n* 7) with a time (*), group (**) or group × time interaction (***) (*P* < 0·05) following a Tukey’s *post hoc* analysis between and within groups.

Metabolites with a significant group, time or group × time effect measured by NMR spectroscopy are presented in Fig. 2. Two large-scale clusters demonstrate two major patterns: an increase in serum concentrations from week 0 to week 12 and a decrease in serum concentrations from week 0 to week 12. These two clusters are broken down further into additional clusters. For serum concentrations dimethylglycine, d-mannose and dimethysulfone, there appears to be a decrease in both groups over time. For serum concentrations of dimethylamine, choline, hypoxanthine, glycine, formate, methanol, l-glutamic acid and acetic acid, there appears to be differences between both CHOLINE and CONTROL from week 0 to week 12. Oxoglutarate, pyruvic acid and l-a-aminobutyric acid serum concentrations appear to increase similarly in both groups from week 0 to week 12. Serum concentrations of l-serine, betaine, methionine and l-threonine are clustered, demonstrating a slight increase in serum concentrations in both CHOLINE and CONTROL. Alternatively, serum concentrations of valine, tyrosine, lysine, l-glutamine and creatinine appear to have a larger increase in CHOLINE than in CONTROL from week 0 to week 12.

### NMR spectroscopy results

Quantitative NMR spectroscopy results are summarised for CHOLINE and CONTROL at week −1 and 12 in Table 8. Methionine increased significantly for CHOLINE (*P*_Group × Time_ = 0·01). Glycine and l-glutamic acid decreased in both groups 12 weeks post-gonadectomy (*P*_Time_ < 0·05). Alternatively, serum concentrations of l-glutamine and valine increased in both groups after 12 weeks (*P*_Time_ < 0·05). l-serine was affected by group (*P*_Group_ = 0·04) and increased for CHOLINE but not for CONTROL after 12 weeks (*P*_Group × Time_ = 0·02). There were no significant differences for amino acids: l-aspartate, l-alanine, l-asparagine, l-arginine and l-proline. No changes or differences were found for l-leucine. However, l-lysine increased in both groups over 12 weeks (*P*_Time_ = 0·01). l-Threonine and tyrosine increased in both groups after 12 weeks (*P*_Time_ = 0·004 and 0·04, respectively). Serum concentrations of l-threonine were different between groups over time (*P*_Group × Time_ = 0·03). No differences or changes were observed in isoleucine or l-phenylalanine between or within groups. Serum concentrations of the augmented amino acid degradation product creatinine increased in both groups (*P*_Time_ = 0·001). No changes or differences were found in serum concentrations of 2-hydroxybutyrate.

**Table 8.** Mean serum metabolite concentrations analysed by quantitative NMR spectrometry in kittens pre-gonadectomy (week −1) and post-gonadectomy (week 12) following supplementation with additional choline at 300 mg/kg BW0·75 (CHOLINE, *n* 8) for 12 weeks compared with a control group (CONTROL, *n* 7) fed only the base diet (3310 mg choline/kg DM)

			CHOLINE (*n* 8)		CONTROL (*n* 7)				
Metabolites (µM)		Week	LSM	sem	LSM	sem	*P*_Group_	*P*_Time_	*P*_Group × time_
Gluconeogenic amino acids	l-Aspartate	–1	58·91	6·86	65·11	6·41	0·69	0·12	0·31
		12	53·87		43·43				
	Glycine	–1	450·85^A^	25·85	490·04^A^	24·10	0·82	0·04	0·08
		12	440·63^B^		388·55^B^				
	l-Alanine	–1	464·17	46·60	466·69	43·49	0·89	0·22	0·83
		12	530·85		514·37				
	l-Arginine	–1	174·02	17·38	214·08	16·20	0·42	0·57	0·18
		12	213·70		197·40				
	l-Asparagine	–1	50·39	5·41	52·17	5·05	0·51	0·06	0·32
		12	66·66		57·64				
	l-Glutamic acid	–1	156·65^A^	6·61	170·87^A^	6·17	0·62	<0·001	0·15
		12	112·65^B^		103·57^B^				
	l-Glutamine	–1	614·68^A^	51·16	719·51^A^	47·66	0·16	0·003	0·51
		12	864·00^B^		891·19^B^				
	l-Proline	–1	130·61	14·51	151·27	13·46	0·76	0·52	0·07
		12	165·00		134·02				
	l-Serine	–1	146·61^A^	12·02	147·17	11·18	0·04	0·21	0·02
		12	190·73^Bc^		132·70^d^				
	Methionine	–1	49·45^A^	4·15	50·84	3·87	0·03	0·008	0·01
		12	73·57^Bc^		51·64^d^				
	Valine	–1	176·71^A^	14·96	191·08^A^	13·95	0·53	0·03	0·11
		12	234·51^B^		200·08^B^				
Ketogenic amino acids	l-Leucine	–1	122·58	40·75	136·19	23·76	0·36	0·08	0·12
		12	224·95		140·75				
	l-Lysine	–1	161·31^A^	15·85	189·85^A^	14·73	0·92	0·01	0·09
		12	231·87^B^		206·82^B^				
Gluconeogenic and ketogenic amino acids	Isoleucine	–1	59·53	4·71	63·69	4·35	0·41	0·27	0·09
		12	72·79		60·73				
	l-Phenylalanine	–1	70·38	7·44	80·77	6·92	0·56	0·06	0·43
		12	90·00		89·23				
	l-Threonine	–1	123·92^A^	13·43	128·24	12·44	0·14	0·004	0·03
		12	194·72^Bc^		141·88^d^				
	Tyrosine	1	47·38^A^	5·71	57·30	5·30	0·47	0·04	0·34
		12	63·82^B^		63·52				
Augmented amino acid degradation products	2-Hydroxybutyrate	–1	13·40	1·54	13·88	1·44	0·47	0·25	0·68
		12	14·58		16·34				
	Creatinine	–1	111·38^A^	10·94	128·48^A^	10·18	0·54	0·001	0·36
		12	165·86^B^		163·38^B^				
Glycolysis	Acetic acid	–1	19·58^A^	1·52	21·55^A^	1·41	0·50	<0·001	0·06
		12	11·77^B^		7·55^B^				
	d-Glucose	–1	15 254	1367·62	17 660	1275·86	0·91	0·17	0·58
		12	16 646		14 510				
	l-Lactic acid	–1	1477·56	162·35	1616·20	151·78	0·09	0·41	0·68
		12	1238·61		1535·24				
Lipolysis	Glycerol	–1	137·72	9·63	129·97	8·99	0·30	0·15	0·07
		12	105·36		133·87				
TCA cycle	Citric acid	–1	235·78	19·91	258·46	18·58	0·08	0·10	0·40
		12	253·12		308·85				
	Malonate	–1	8·98	0·83	10·08	0·78	0·53	0·80	0·14
		12	10·75		8·82				
	Oxoglutarate	–1	3·34^A^	1·18	3·12^A^	1·18	0·60	0·04	0·48
		12	5·31^B^		6·81^B^				
	Pyruvic acid	–1	10·51^A^	2·88	9·28^A^	2·69	0·59	0·002	0·34
		12	18·42^B^		22·77^B^				
	Succinate	–1	1·62	0·26	1·94	0·24	0·87	0·28	0·28
		12	2·17		1·94				
Ketogenesis	Acetone	–1	246·64	92·18	197·30	86·22	0·27	0·22	0·97
		12	102·32		42·41				
	Acetoacetate	–1	0·53	0·12	0·49	0·11	0·59	0·15	0·97
		12	0·75		0·69				
One-carbon metabolism	Betaine	–1	165·19^A^	44·42	205·74	41·34	0·02	0·002	0·001
		12	499·21^Bc^		191·14^d^				
	Choline	–1	19·28	1·62	19·55^A^	1·52	0·61	0·02	0·68
		12	15·08		13·68^B^				
	Creatine	–1	15·24	1·89	15·97	1·53	0·43	0·53	0·22
		12	18·48		14·95				
	Formate	–1	26·87^A^	0·69	26·90^A^	0·64	0·97	<0·001	0·99
		12	20·10^B^		20·12^B^				
	Dimethylglycine	–1	7·92^A^	0·68	8·10	0·64	0·001	0·03	<0·001
		12	12·82^Bc^		6·35^d^				
	l-Carnitine	–1	9·30	1·43	9·18	1·34	0·44	0·64	0·40
		12	8·74		11·05				
Purine degradation	Hypoxanthine	–1	14·74	2·61	14·34	2·44	0·002	0·03	0·59
		12	4·14		7·66				
Alcohols	Methanol	–1	470·06^A^	56·89	474·79^A^	53·21	0·19	0·001	0·21
		12	175·94		325·98				
	Ethanol	–1	52·77	11·57	66·58	10·82	0·18	0·97	0·78
		12	57·37		63·14				
	Isopropanol	–1	478·59	122·35	518·92	114·44	0·14	0·05	0·43
		12	271·88		57·01				
	Propylene glycol	–1	121·26	15·67	129·84	13·59	0·35	0·07	0·71
		12	85·93		105·65				
Other	d-Mannose	–1	62·71^A^	5·38	65·21^A^	5·03	0·04	0·09	0·17
		12	60·17^c^		43·24^Bd^				
	l-Acetyl carnitine	–1	2·59	0·27	2·64	0·25	0·75	0·80	0·61
		12	2·66		2·44				
	Urea	–1	2462·08	109·69	2446·86	102·60	0·31	0·94	0·25
		12	2326·76		2566·73				
	2-Hydroxyisovalerate	–1	3·37	0·38	3·68	0·35	0·50	0·86	0·11
		12	4·03		3·14				
	l-*α*-Aminobutyric acid	–1	3·43^A^	1·85	3·62^A^	1·73	0·56	0·02	0·49
		12	9·42^B^		7·07^B^				
	3-Methyl-2-oxovaleric acid	–1	2·51	0·20	2·51	0·18	0·43	0·46	0·42
		12	2·81		2·50				
	Dimethylsulfone	1	5·81^A^	0·37	5·99^A^	0·35	0·27	0·04	0·26
		12	5·33^B^		4·51^B^				
	Ketoleucine	–1	2·61	0·28	2·48	0·26	0·36	0·17	0·62
		12	3·13		2·73				
	3-Hydroxyisovaleric acid	–1	1·42	0·26	1·29	0·24	0·08	0·68	0·58
		12	1·73		1·24				
	Dimethylamine	–1	3·30^A^	0·16	3·27^A^	0·15	0·77	0·02	0·89
		12	2·90^B^		2·84^B^				

LSM, least square means.

Rows or columns with no superscript indicates no significant differences for the measured parameter.

Down a column, different upper-case superscript letters (A, B) denote significant differences (*P* < 0·05) following a Tukey’s *post hoc* analysis within group (CHOLINE or CONTROL) over time (week −1 to week 12).

Across a row, different lower-case letters (c, d) denote significant differences (*P* < 0·05) following a Tukey’s *post hoc* analysis between groups (CHOLINE and CONTROL) at one time point (week −1 or week 12).

Metabolites of glycolysis and lipolysis were also analysed by NMR spectroscopy (Table 8). Acetic acid concentrations decreased for both CHOLINE and CONTROL 12 weeks post-gonadectomy (*P*_Time_ < 0·001). No changes or differences were observed in d-glucose and l-lactic acid serum concentrations between or within groups (*P* > 0·05). No changes were found in the lipolysis metabolite, glycerol.

Serum concentrations of metabolites of the TCA cycle, oxoglutarate and pyruvic acid increased in both groups post-gonadectomy (*P*_Time_ = 0·04 and 0·002, respectively). Concentrations of citric acid, malonate and succinate were not different between or within groups. Furthermore, serum concentrations of acetone and acetoacetate did not change over time within groups nor did they differ between groups.

Choline supplementation had a significant effect on betaine and dimethylglycine (*P*_Group_ = 0·02 and 0·001, respectively). Serum betaine concentrations increased after 12 weeks for CHOLINE, whereas they decreased for CONTROL (*P*_Time_ = 0·002 and *P*_Group × Time_ = 0·001). Similarly, serum concentrations of dimethylglycine increased for CHOLINE 12 weeks post-gonadectomy, whereas serum dimethylglycine concentrations were decreased for CONTROL (*P*_Time_ = 0·03 and *P*_Group × Time_ < 0·001). Serum choline and formate concentrations decreased in both groups regardless of group after 12 weeks (*P*_Time_ = 0·02 and *P*_Time_ < 0·001, respectively). No changes or differences were observed in creatine or l-carnitine concentrations.

Serum concentrations of hypoxanthine decreased in both groups after 12 weeks (*P*_Time_ = 0·03); however, choline appeared to have an effect, resulting in lower serum concentrations of hypoxanthine (*P*_Group_ = 0·002). No changes or differences were observed within or between groups for the alcohol’s ethanol, isopropanol and propylene glycol. However, methanol appeared to decrease in both groups regardless of treatment.

Serum concentrations of d-mannose decreased for CONTROL; however, choline supplementation did not change serum concentrations of d-mannose (*P*_Group_ = 0·04). Serum l-*α*-aminobutyric acid concentrations increased, whereas dimethylsulfone and dimethylamine concentrations decreased, in both groups regardless of group 12 weeks post-gonadectomy (*P*_Time_ = 0·02, 0·04 and 0·02, respectively). No changes or differences were observed in the following metabolites between or within groups: l-acetyl carnitine, urea, 2-hydroxyisovalerate, 3-methyl-2-oxovaleric acid, ketoleucine and 3-hydroxyisovaleric acid.

## Discussion

As hypothesised, daily choline supplementation at 300 mg/kg BW^0·75^, resulting in four times the recommended minimum choline requirement for growth according to the National Research Council (NRC)^(37)^, had a significant effect on the serum metabolic profiles of kittens after neutering. In addition, the results from the present study indicate changes in the serum metabolome of kittens over time. However, due to constraints in the present study design, it is unclear whether these changes are due to growth or gonadectomy. As previously published, voluntary energy intake for CHOLINE and CONTROL was 2 % and 18 % greater than energy requirements^(34)^. The mean total choline intakes for CHOLINE was 1146·60 (±21·01) mg/d and 287·40 (±22·46) mg/d for CONTROL^(34)^. Kittens in both groups were expected to gain BW though the CHOLINE group gained significantly less BW than CONTROL; CHOLINE had a 26 % increase in BW from pre-neuter to 12 weeks post-neuter, while CONTROL experienced a 38 % increase in BW(34). From dual-energy X-ray absorptiometry, on average, kittens in the CHOLINE group at 12 weeks post-neuter had lower mean body fat mass (1·36 ± 0·04 kg) and a body fat percent of 29 % compared with CONTROL (1·67 ± 0·04 kg and 34 %, respectively), though lean soft tissue mass was not affected by choline supplementation^(34)^.

Surprisingly, additional choline did not affect circulating serum concentrations of choline. However, this aligns with the findings in Pcyt-heterozygous mice, growing mink kits and adult mink^(33,45,46)^. Circulating levels of choline are not good indicators of choline status, as choline is quickly utilised after intestinal absorption either by oxidation to betaine^(45)^ or phosphorylation to PC^(46)^. Similar to growing mink kits, choline supplementation in the present study increased serum concentrations of betaine in growing kittens. This is further evidence that choline is quickly metabolised and thereby may not be accurately reflected in extracellular concentrations. Betaine is a methyl donor for one-carbon metabolism. An increase in dimethylglycine with choline supplementation, as observed here, suggests higher betaine homocysteine S-methyltransferase activity, remethylating homocysteine to methionine. This is further supported by the increase in serum methionine concentrations in CHOLINE compared to CONTROL.

Choline and betaine metabolism occur in the mitochondria and relies on products from the TCA cycle^(47)^. Choline dehydrogenase, which oxidizes choline to betaine aldehyde, uses flavin adenine dinucleotide (FADH) and produces FADH_2_, as well as ATP. Whereas demethylation of betaine uses NADH. This relationship between choline and betaine oxidation and the TCA cycle was observed in Pcyt2-deficient mice, in which an increased demand for the TCA cycle was observed^(2)^. However, in the current study, additional choline did not appear to upregulate serum metabolites of the TCA cycle, though it is important to note that serum metabolites of TCA may not provide adequate insight to the intracellular activity of the TCA cycle.

It appears that one-carbon metabolism was increased by additional choline due to the increase in betaine, dimethylglycine, l-serine and methionine. Dimethylglycine is then metabolised to sarcosine^(48)^, which also increased with CHOLINE. It is hypothesised that with an increase in choline-betaine-driven methionine re-methylation, there was a reduced demand for the folate cycle to support methionine re-methylation. The increase in serine concentrations with no changes in serum glycine concentrations support this hypothesis, suggesting a decrease in serine-hydroxymethyltransferase activity in which serine is converted to glycine as part of the folate cycle. However, the serum serine, sarcosine and glycine pool are too large to make definitive conclusions.

The increased serum methionine concentrations in kittens on CHOLINE may provide some insight into potential hypotheses with regard to protein synthesis and transmethylation reactions. Methionine can be partitioned either to protein or to S-adenosylmethionine (SAM)^(49)^. During growth specifically, the requirements for methionine are substantial and create a significant burden on methionine^(50,51)^. Methionine as SAM is used for the synthesis of creatine, carnitine and PC, all of which may be required at greater levels during growth^(50,51)^.

Arginine and glycine are used for the formation of guanidinoacetate and ornithine. Guanidinoacetate with SAM forms creatine, as well as S-adenosylhomocysteine^(52)^. In neonatal pigs, creatine synthesis activity was high, alongside an increased demand for methionine re-methylation^(53)^. Creatine synthesis resulted in a burden on methionine metabolism. It was thought that by increasing the re-methylation capabilities of the one-carbon metabolism, methionine would be more readily available, and the nutritional burden would be reduced; therefore, it was hypothesised that creatine synthesis could be increased in kittens with additional choline. However, compared to kittens on CONTROL, additional choline did not appear to alter serum concentrations of arginine, glycine, ornithine or creatine. While this does not provide support for the hypothesis for creatine synthesis, it does not eliminate this possibility, as serum metabolites such as creatine are poor indicators of intracellular creatine status. Skeletal muscle creatine content may provide a deeper understanding of how methionine, via one-carbon metabolism, could affect creatine concentrations. Interestingly, serum creatinine, a product of creatine, was increased over time in the serum of growing kittens, which is likely due to the increased lean soft tissue mass in these kittens^(34)^, as excretion of creatinine is generally in proportion to muscle mass^(54)^.

All kittens consumed above their energy requirement, and subsequently, their amino acid requirements^(34)^ according to the NRC minimum nutrient requirements predictions, and had fasted serum amino acid concentrations within or above previously documented concentrations^(55–58)^. Methionine intake was 37·5 % greater for CHOLINE and 50 % greater for CONTROL compared to the recommended NRC^(37)^ minimum requirements for growing kittens. Importantly, mean daily amino acid intake was greater for CONTROL than for CHOLINE due to greater food consumption^(34)^. Higher serum amino acid concentrations over the course of the experimental period in both groups would be expected due to growth and lean soft tissue mass development^(59)^. While growth in female kittens appears to slow by 10 months of age, male kittens appear to have steady growth of lean and fat mass as well as bone mineral content and density until 1 to 2 years of age, reaching adult BW by approximately 16 months of age and, therefore, protein deposition and turnover may be greater in contrast to their female counterparts^(60–62)^. Circulating concentrations of alanine, arginine, asparagine, glutamate, leucine, lysine, methionine, proline, serine, threonine and valine in neonatal pigs are indicative of muscle protein synthesis and lean tissue mass maintenance^(44,47)^. In the present study, increases over time in fasted serum concentrations of asparagine, glutamine, lysine, methionine and threonine, independent of group, could be attributed to growth, development and maintenance of lean soft tissue as total mass increases with age.

As described previously, choline may act to increase protein deposition in growing male kittens via SAM. In neonatal pigs, the importance of methionine, and other methyl donors (choline, betaine and folate), for muscle and protein synthesis has been consistently documented^(63–65)^. Furthermore, in insulin-resistant, Pcyt2-deficient mice, choline supplementation promoted an increase in skeletal muscle protein synthesis and metabolism^(66)^. In growing kittens, it is impossible to attribute choline supplementation to improved protein deposition. In these kittens, the lean soft tissue mass in both CHOLINE and CONTROL were not different^(34)^. It could be that if choline increases re-methylation of methionine via one-carbon metabolism in the CHOLINE group, who were consuming significantly less amino acids than CONTROL, then there is less burden to methionine resulting in higher partitioning of methionine to protein deposition. However, since all kittens exceeded amino acid and energy requirements according to the recommendations of the NRC^(37)^, protein synthesis was likely maximised^(67)^. Future studies should consider assessing the effects of additional choline in kittens during growth when food intake is controlled for.

A similar approach may be necessary to further understand the impact of choline on other pathways, such as gluconeogenesis. Cats, as obligate carnivores, rely heavily on alanine, asparagine, arginine, glutamine, glutamate and serine as amino acids for gluconeogenesis to maintain blood glucose levels^(68)^. In the present study, fasted serum concentrations of asparagine and glutamine increased, which could likely be an effect of growth, gluconeogenesis and glucose capacity^(69)^. As kittens reach maturity, amino acids requirements decrease as protein deposition decreases. However, as amino acid intakes did not decrease, amino acids were likely oxidised, increasing the nitrogen-carrying requirement, which may have been fulfilled by glutamine in the kittens, resulting in the increase in serum concentrations. Interestingly, although CHOLINE had lower amounts of these amino acids^(34)^, there were no differences in the fasted serum concentrations of asparagine and glutamine between groups. It may be that additional dietary choline can impact these concentrations; however, similar to the methionine partitioning and protein deposition hypotheses, amino acid intakes would need to be controlled for in future studies to explore this hypothesis. Future studies using isotope dilution techniques may also be used to quantify the rate of gluconeogenesis in response to choline supplementation to further understand these roles of choline. The greater serine in CHOLINE may also be attributed to the mechanisms described above in response to greater choline metabolism.

Insulin-resistant mice with additional choline supplementation (10 mg choline/d) showed greater markers of fatty acid oxidation than insulin-resistant mice without additional choline after 4 weeks of treatment^(66)^. Supplementation of choline upregulated AMPK and restored dysregulation of mTORC1. Fatty acid oxidation is stimulated by AMPK, whereas lipogenesis is downregulated. In this study, the activities of AMPK and mTORC1 were not analysed; however, a decrease in serum medium- and long-chain acyl carnitines have been associated with improved and increased fatty acid oxidation, while an increase is associated with metabolic dysfunction and obesity in humans and mice^(70,71)^. In the present study, there was a decrease in serum medium-chain acyl carnitines in the CHOLINE group compared to CONTROL. This may further support the hypothesis that additional choline may have beneficial effects on fatty acid oxidation and thus reduction of adipose deposition. With time, there appeared to be an increase in certain acyl carnitines in both groups. However, this could be due to growth and not due to changes in fatty acid oxidation. The higher fasted serum PC concentrations observed with choline supplementation could also indicate a shift towards fatty oxidation rather than lipogenesis and could be beneficial in preventing the weight and body fat mass gains associated with gonadectomy.

The effects of additional choline on greater serum concentrations of glycerol-phospholipids, such as PC and LPC, were observed. Higher serum concentrations have been associated with benefits for prevention and treatment of obesity^(72–74)^. A large proportion, up to 90 %, of available choline, is converted to PC via the CDP-choline pathway, also known as the Kennedy pathway^(75)^. Furthermore, the PEMT pathway synthesises PC from phosphatidylethanolamine via SAM, and this can be a significant burden on methionine requirements as three methyl groups, or three molecules of SAM, are required for this pathway. It is likely that kittens with CHOLINE reduced the requirement of SAM for the PEMT pathway as the CDP-choline pathway may have had a higher activity; however, this cannot be confirmed. Therefore, as was expected, additional choline increased fasting serum concentrations of PC in kittens supplemented with additional choline.

PC lowered bile and hepatic concentrations of cholesterol and TAG in VLDL in 3-month-old rabbits with hyperlipidemia^(73)^ and attenuated induced fatty liver in Sprague-Dawley rats^(74)^. In growing, 4-week-old mice, PC reduced hepatic TAG synthesis and increased fatty acid oxidation^(72)^. These results are promising, though comparisons for cats, rabbits and mice may not be accurate due to the nutritional idiosyncrasies of the cat. Lipid metabolism in rabbits and mice are more closely related to humans than to cats^(76)^.

When PC are cleaved by phospholipases as a result of LDL oxidation, LPC is formed^(77)^. With an increase in serum PC concentrations, it was expected that serum LPC concentrations would also increase with choline supplementation. The relationship between LPC concentrations and health status with regard to obesity and the metabolic syndrome remain unclear^(78)^. Similarly, SM concentrations may be indicative of obesity and other health complications^(79)^. SM require choline and are incorporated into cell membranes^(80)^. In humans, there appears to be some consistency in lipidomic and metabolic profiles that certain LPC are decreased in obese models^(61,64–66)^, whereas serum SM concentrations are increased in human CVD^(35)^. From these metabolic profiles, the human obese model experiences increased saturated LPC, such as LPC C16:0, which exerts a pro-inflammatory effect; however, unsaturated LPC concentrations (C18:1, C18:2 and C20:4) are decreased. Similar findings have been demonstrated in human adolescence and obesity^(81–83)^. Kittens were in the growth stage and were not considered obese, and therefore, direct comparisons to obese models may be difficult. Interestingly, kittens with additional dietary choline experienced some trends for greater SM concentrations; however, these were unsaturated SM and may not be indicative of inflammatory responses or obesity onset. Further understanding of SM and their role during growth and weight management may be warranted. In addition, current data are limited to humans, omnivores, and the role of SM may differ in the cat, an obligate carnivore. Therefore, these potential differences should be further investigated to deepen our understanding.

Overall, the use of Di-MS and NMR mass spectroscopy to determine the metabolic profile of kittens supplemented with choline for 12 weeks post-gonadectomy, in the present study, demonstrated that additional dietary choline may be beneficial in mitigating the negative consequences associated with *ad libitum* food intake, and gonadectomy. From the results of the present study, there appears to be a shift towards increased fatty acid oxidation, which would decrease lipogenesis, theoretically resulting in less body fat mass in the growing kittens and, potentially, providing a protective effect against obesity onset commonly seen with gonadectomy. Additional researchers presented earlier that dietary choline did result in less body fat mass after 12 weeks resulting in less total tissue mass^(34)^, further perpetuating this hypothesis. Previous studies have concluded an increase in lipolysis alongside a decrease in lipogenesis^(27,28,84)^; however, in growing kittens, it is unlikely that there is sufficient adipose tissue to see changes in lipolysis.

The present study is limited as it is only able to report on changes of the serum metabolic signature after additional choline supplementation and lacks tissue-specific metabolomics and/or genomics. Serum concentrations of metabolites provides opportunities to build specific hypotheses, but due to the large pools of many metabolites from differing pathways and the inability to determine the location of output and uptake by different tissues, this study is limited to generating hypotheses only and future, targeted hypothesis-driven studies should be considered for future research. There may be benefits to understanding changes within tissue concentrations of metabolites, specifically those relevant to one-carbon metabolism and fatty acid oxidation, to further investigate changes in tissues such as the liver and muscle, which may also help elucidate where metabolites are produced to further understand the metabolic changes. It has consistently been found that choline supplementation results in greater protein synthesis and turnover of skeletal muscle^(85)^. Evaluating the muscle content of mTORC1 and AMPK, as well metabolites such as creatine and carnitine, may also provide more insights into the benefits of choline for optimal growth and preventing potential obesity risk factors from gonadectomy.

Another limitation is the mimicked *ad libitum* feeding strategy utilised in this study, which resulted in different energy and amino acid intake between groups^(34)^. Future studies may consider assessing changes in the metabolic signature when energy and amino acid intake is controlled for through pair-feeding may help to evaluate the effects of dietary choline without differences in food and dietary energy intake. The present study also attempts to make assumptions on the effects of gonadectomy on the metabolic signature; however, the lack of an intact control group and a lack of existing literature on growing kittens, gonadectomy and metabolic signatures limits the ability to make direct correlations. While we acknowledge these limitations, it is important to consider the difficulty in maintaining an intact male cat cohort

Overall, dietary choline should be considered as a potential nutrient for the up-regulation of one-carbon metabolism. Furthermore, its impacts on PC and its subsequent metabolites could be indicative of improved fatty acid mobilisation and metabolism during growth and after gonadectomy procedures. Additional dietary choline should be further investigated as a potential nutritional strategy to combat weight gain and obesity onset in cats when exposed to obesity risk factors.

## Supplementary material

For supplementary material accompanying this paper visit https://doi.org/10.1017/S0007114522003385. click here to view supplementary material
